# Spatial Distribution of, and Risk Factors for, *Opisthorchis viverrini* Infection in Southern Lao PDR

**DOI:** 10.1371/journal.pntd.0001481

**Published:** 2012-02-14

**Authors:** Armelle Forrer, Somphou Sayasone, Penelope Vounatsou, Youthanavanh Vonghachack, Dalouny Bouakhasith, Steffen Vogt, Rüdiger Glaser, Jürg Utzinger, Kongsap Akkhavong, Peter Odermatt

**Affiliations:** 1 Department of Epidemiology and Public Health, Swiss Tropical and Public Health Institute, Basel, Switzerland; 2 University of Basel, Basel, Switzerland; 3 National Institute of Public Health, Ministry of Health, Vientiane, Lao PDR; 4 Faculty of Basic Sciences, University of Health Sciences, Vientiane, Lao PDR; 5 Geographical Institute, University of Freiburg, Freiburg im Breisgau, Germany; London School of Hygiene & Tropical Medicine, United Kingdom

## Abstract

**Background:**

*Opisthorchis viverrini* is a food-borne trematode species that might give rise to biliary diseases and the fatal cholangiocarcinoma. In Lao PDR, an estimated 2.5 million individuals are infected with *O. viverrini*, but epidemiological studies are scarce and the spatial distribution of infection remains to be determined. Our aim was to map the distribution of *O. viverrini* in southern Lao PDR, identify underlying risk factors, and predict the prevalence of *O. viverrini* at non-surveyed locations.

**Methodology:**

A cross-sectional parasitological and questionnaire survey was carried out in 51 villages in Champasack province in the first half of 2007. Data on demography, socioeconomic status, water supply, sanitation, and behavior were combined with remotely sensed environmental data and fed into a geographical information system. Bayesian geostatistical models were employed to identify risk factors and to investigate the spatial pattern of *O. viverrini* infection. Bayesian kriging was utilized to predict infection risk at non-surveyed locations.

**Principal Findings:**

The prevalence of *O. viverrini* among 3,371 study participants with complete data records was 61.1%. Geostatistical models identified age, Lao Loum ethnic group, educational attainment, occupation (i.e., rice farmer, fisherman, and animal breeder), and unsafe drinking water source as risk factors for infection. History of praziquantel treatment, access to sanitation, and distance to freshwater bodies were found to be protective factors. Spatial patterns of *O. viverrini* were mainly governed by environmental factors with predictive modeling identifying two different risk profiles: low risk of *O. viverrini* in the mountains and high risk in the Mekong corridor.

**Conclusions/Significance:**

We present the first risk map of *O. viverrini* infection in Champasack province, which is important for spatial targeting of control efforts. Infection with *O. viverrini* appears to be strongly associated with exposure to the second intermediate host fish, human behavior and culture, whereas high transmission is sustained by the lack of sanitation.

## Author Summary

The liver fluke *Opisthorchis viverrini* mainly occurs in Lao PDR and Thailand. Humans become infected through the consumption of raw or insufficiently cooked freshwater fish. Chronic infections may lead to severe liver (bile duct) diseases that eventually develop into a bile duct cancer with extremely poor prognosis. Current control efforts aim at preventing heavy morbidity and mortality. In recent years, spatial modeling, using data from well designed surveys, has been employed to better understand the distribution and determinants of parasitic diseases for guiding subsequent control. However, a spatial modeling approach has not been used for *O. viverrini* before. The purpose of the current study was to map the distribution of *O. viverrini* infection in Champasack province in southern Lao PDR, to identify risk factors of infection, and to predict the distribution at non-surveyed locations. We found that the risk of *O. viverrini* infection is higher for people living in close proximity to freshwater bodies, whereas the lack of sanitation sustained environmental contamination and transmission. High risk zones in Champasack province are concentrated in the Mekong River corridor, and hence control efforts should be targeted along the Mekong River.

## Introduction

The liver fluke *Opisthorchis viverrini* is of considerable public health importance in Southeast Asia, particularly in Lao PDR and Thailand [1], [2]. Indeed, it has been estimated that 85% of the 10 million *O. viverrini* infections are concentrated in those two countries [3], [4]. For the mid-1990s, when the overall population in Lao PDR was approximately 5 million people (Lao Statistics Bureau, Vientiane; http://www.nsc.gov.la), half of them were thought to be infected with *O. viverrini* [4]. Highest infection rates were observed in the centre and in the southern provinces of Lao PDR [5]–[9]. However, recent mapping efforts are lacking, although accurate maps are a prerequisite for efficient targeting of scarce resources for control. Chronic *O. viverrini* infection is related to a number of severe liver and bile duct diseases, such as cholecytitis, cholangitis, and periductal fibrosis [1], [2], [8], [10]. Most importantly, *O. viverrini* is a major risk factor for cholangiocarcinoma (CCA), a bile duct cancer with extremely poor prognosis [2], [11], [12].

Among other issues, the spatial distribution of parasitic diseases is governed by environmental factors that determine ecological niches for the parasite and their vectors and/or intermediate hosts [13]–[15]. Regarding *O. viverrini*, there are two freshwater intermediate hosts implicated in its life cycle: snails of the genus *Bithynia* are the first intermediate host, whereas Cyprinidae fish serve as the second intermediate host [16]. Human acquire an infection through consumption of raw or insufficiently cooked cyprinoid fish. Open defecation results in contaminated water bodies. The transmission between intermediate hosts mainly depends on the temperature of freshwater bodies and the amount and duration of rainfall [17]. Geospatial statistics have proven useful to study the distribution and underlying risk factors for parasitic diseases, with environmental factors found to be important determinants of helminth infections such as schistosomes and hookworm [14], [17]–[22]. However, to our knowledge, a geostatistical modeling approach has yet to be employed to further the understanding of the spatial distribution of, and risk factors for, *O. viverrini* infection.

The purpose of the current study was to map and predict the distribution of *O. viverrini* infection in a southern province of Lao PDR where opisthorchiasis is highly endemic. Particular emphasis was placed on the relative importance of environmental risk factors in comparison to demographic, socioeconomic, water, sanitation, and behavioral determinants.

## Methods

### Ethics Statement

The study was approved by the institutional research commission of the Swiss Tropical and Public Health Institute (Swiss TPH; Basel, Switzerland). Ethical approval was obtained from the Ministry of Health (MoH) of Lao PDR (reference no. 027/NECHR) and the ethics committee of Basel (EKBB; reference no. 255/06), which also approved the informed consent procedure. Permission for field work was obtained from MoH, the Provincial Health Office (PHO), and the District Health Office (DHO). Village meetings were held and local authorities and inhabitants were given detailed explanations about the aims, procedures, potential risks, and benefit of the study. The information sheet in the local language was read to all household members and their questions answered. Individual oral consent was obtained from all adult household members (literacy is very low in this part of Lao PDR, and hence we opted for oral consent). Additionally, written informed consent was obtained from all heads of households. A witness observing this procedure also signed the consent form. Participants found positive for intestinal parasites were treated according to national guidelines [23]. Those infected with *O. viverrini* were treated with praziquantel at a single oral dose of 40 mg/kg.

### Study Area

The study was carried out in Champasack, the largest province of southern Lao PDR. According to the 2005 census, the total population of Champasack is 603,370 (Census 2005, Lao Statistics Bureau, Vientiane; http://www.nsc.gov.la). Run through from North to South by the Mekong River, the province covers an area of 15,415 km^2^, stretching from 13°55′ to 15°29′N latitude and 105°11′ to 106°46′E longitude. In terms of ecology, two-third of the province is characterized by plains and flatland dominated by the Mekong River, whereas the north-east is mountainous, with an elevation of up to 1,617 m above sea level. The climate is of monsoon tropical type, with the rainy season occurring between May and October.

Intestinal parasitic infections are common in the Mekong River basin. Specifically, *O. viverrini* and other fluke infections (i.e., minute intestinal flukes (MIF) and the blood fluke *Schistosoma mekongi*), and soil-transmitted helminths, are endemic [9], [24]–[27].

### Cross-Sectional Epidemiological Survey

Epidemiological data were obtained from a cross-sectional community-based survey carried out between January and May 2007 in all nine rural districts of Champasack province, whereas the tenth district being urban was excluded. Overall, 4,380 individuals in 51 villages were selected. Sample selection was achieved using a two-stage sampling method: first a random selection of villages and, second, a random selection of 10–15 households per village. All individuals aged 6 months and above were included. Household coordinates were recorded using a hand-held global positioning system (GPS) receiver (Garmin Ltd.; Olathe, United States).

### Demographic, Behavioral, and Socioeconomic Data

Demographic (e.g., age, sex, and educational attainment) and behavioral data (e.g., food consumption and hygiene habits), including medical history, were obtained from each participant using a questionnaire. For children aged <10 years, parents or legal guardians were interviewed. Additionally, a household questionnaire was administered to the heads of household to collect socioeconomic data, such as access to clean water and sanitation, house construction material, and ownership of household assets and livestock.

### Parasitological Data

Study participants were invited to provide a single stool sample. Duplicate Kato-Katz thick smears, using 41.7 mg templates, were prepared shortly after stool collection (within a maximum of 2 hours), following a standard protocol [28]. Kato-Katz thick smears were allowed to clear for 30 min before examination under a microscope by experienced laboratory technicians. Helminth eggs were counted and recorded for each species separately. For quality control, 10% of the Kato-Katz thick smears were re-examined by a senior technician. Helminth-specific egg counts were compared with the original readings and, whenever discrepancies were observed, the slides were read a third time until agreement was reached.

### Environmental Data

Environmental parameters were readily extracted from freely available remote sensing (RS) sources at temporal and spatial resolutions summarized in Table S1. Enhanced vegetation index (EVI), day and night land surface temperature (LST), and land use/land cover (LULC) consisting of 18 land cover type 1 classes (IGBP), were downloaded from Moderate Resolution Imaging Spectroradiometer (MODIS) Land Processes Distributed Active Archive Center (LP DAAC), United States Geological Survey (USGS) Earth Resources Observation and Science (EROS) Center (http://lpdaac.usgs.gov). Rainfall estimates (RFE) were obtained from CPC FEWS Rainfall Estimates South Asia version 2.0 (http://www.cpc.ncep.noaa.gov/products/fews/SASIA/rfe.shtml). Digital elevation data were retrieved from the NASA Shuttle Radar Topographic Mission (SRTM) and CGIAR-CSI database, whereas distance to large water bodies was obtained from Health Mapper. LULC 18 classes were merged into four categories according to similarity and respective frequencies. Seasonal means of EVI, monthly LST and RFE were calculated for May to October 2006 and November 2006 to April 2007.

### Statistical Analysis

#### Data management

Survey data were double-entered using EpiData 3.1 (EpiData Association; Odense, Denmark) and cross-checked. Environmental data processing, georeferencing, and maps were made in ArcGIS version 9.2 (ESRI; Redlands, CA, United States). Environmental data were linked to parasitological and questionnaire data according to georeferenced location. Database processing and non-spatial statistical analysis were performed in STATA version 10 (StataCorp LP; College Station, United States). Age was categorized into five classes, as follows: (i) <4 years, (ii) 5–17 years, (iii) 18–39 years, (iv) 40–59 years, and (v) ≥60 years. Original categories of variables with frequencies under 5% were merged with similar categories.

#### Socioeconomic status

House construction material and ownership of household assets were employed to build an asset-based socioeconomic index, using multiple correspondence analysis (MCA), a data reduction technique that has been developed for categorical data [29], [30]. Households were classified into five wealth quintiles, the first quintile corresponding to the most poor and the fifth to the least poor.

#### Statistical tests

Chi-square (χ^2^) test was used to compare proportions. The association between infection risk and covariates was assessed, using non spatial bivariate logistic regressions. Covariates exhibiting an association at a significance level of at least 15%, as determined by the likelihood ratio test (LRT), were included in the multivariate logistic regression models. In case of correlation, the variable resulting in the model with the smallest Akaike's information criterion (AIC) was selected. Dry season means for EVI, LST day, LST night, and RFE were standardized before inclusion in the multiple regression models.

#### Geostatistical models

Logistic geostatistical models were fitted using WinBUGS version 1.4.3 (Imperial College & Medical Research Council; London, United Kingdom). A stationary isotropic process was assumed, with village-specific random effects following a normal distribution with mean zero and a variance-covariance matrix being an exponential function of the distance between pairs of locations. Non-informative prior distributions were chosen for all parameters. Further information is provided in Appendix S1. Results were not sensitive to changes in the prior boundaries. Due to the low frequency of people who received praziquantel (3.5%), an alternative model was run on a subsample, in which participants for whom treatment was unsure or unknown were excluded. This subset constituted of 2,280 individuals, and all 51 villages were represented. Prevalence and proportions for all covariates were similar to those of the total sample.

Spatial models, together with their non-spatial counterparts, were run with various combinations of covariates, as well as one spatial model without covariates. Model specifications are detailed in Table S2. Markov chain Monte Carlo (MCMC) simulation was used to estimate model parameters [31]. Convergence was assessed through examination of the ergodic averages of selected parameters. Before drawing samples, it was verified for each parameter that the MC error was below 5% of the standard deviation [32]. The fitting of various models was appraised with the deviance information criterion (DIC); the lower the DIC, the better the model fit [33].

#### Prediction of *O. viverrini* infection at non-surveyed locations

The risk of *O. viverrini* infection at non-surveyed locations was predicted using Bayesian kriging [34]. Predictions were made based on environmental factors only (model 2), with the WinBUGS “spatial.unipred” function [32] at 15,156 pixels of 1×1 km spatial resolution. Model fit was run on a random subset of 41 villages, whereas the remaining 10 villages were used as test locations. For model validation, the predictive ability of the model was assessed using the probability coverage of the shortest Bayesian credible interval that included the largest number of test locations [35].

## Results

### Adherence and Final Cohort

A total of 4,380 individuals were invited to participate, among whom 280 were absent during registration, 709 missed either the parasitological or the questionnaire survey, and 20 had incomplete questionnaire data. Hence, the final sample consisted of 3,371 individuals (76.9%) from 815 households in 51 villages. Table 1 summarizes the key features of our study cohort. In brief, there were significantly more female than male participants (52.7% *vs.* 47.3%). The median age was 21.2 years with an inter-quartile range of 32.4 years. The percentage of individuals aged ≤4, 5–17, 18–39, 40–59 and ≥60 years was 9.6%, 36.6%, 26.3%, 19.3% and 8.2%, respectively. Most of the participants belonged to the Lao Loum ethnic group (81.7%). Over three-quarter of the respondents (76.3%) did not have access to any kind of sanitation and 27.4% (14/51) of the villages had no toilet facilities at all. None of the villages surveyed had complete sanitation coverage.

**Table 1:** Characteristics of total sample and subsample (after adjustment for praziquantel treatment history).

Variable	Category	Total sample (n = 3,371)	Subsample (n = 2,280)
		n (%)	n (%)
**Sex**	Female	1,776 (52.7)	1,213 (53.2)
	Male	1,595 (47.3)	1,067 (46.8)
**Ethnic group**	Other	616 (18.3)	398 (17.5)
	Lao Loum	2,755 (81.7)	1,882 (82.5)
**Age (years)**	≤4	325 (9.6)	258 (11.3)
	5–17	1,235 (36.6)	742 (32.5)
	18–39	887 (26.3)	623 (27.3)
	40–59	649 (19.3)	462 (20.3)
	≥60	275 (8.2)	195 (8.6)
**Main occupation**	No occupation	732 (21.7)	564 (24.7)
	School pupil	897 (26.6)	472 (20.7)
	University, employee, business	88 (2.6)	59 (2.6)
	Rice farmer	1,339 (39.7)	965 (42.3)
	Fisherman, animal breeder, other	315 (9.3)	220 (9.7)
**Socioeconomic status**	Most poor	582 (17.3)	434 (19)
	Very poor	657 (19.5)	426 (18.7)
	Poor	739 (21.9)	482 (21.1)
	Less poor	715 (21.2)	470 (20.6)
	Least poor	678 (20.1)	468 (20.5)
**Education level**	Illiterate	965 (28.6)	736 (32.3)
	Primary school	1,767 (52.4)	1,085 (47.6)
	Secondary school and higher	639 (19.0)	459 (20.1)
**Consumption of raw or undercooked fish**	No	1,951 (57.9)	1,312 (57.5)
	Yes	1,420 (42.1)	968 (42.5)
**Consumption of fermented fish**	No	872 (25.9)	607 (26.6)
	Yes	2,499 (74.1)	1,073 (73.4)
**Consumption of bottled or boiled drinking water**	No	1,518 (45.0)	1,076 (47.2)
	Yes	1,853 (55.0)	1,204 (52.8)
**Access to toilets**	No	2,572 (76.3)	1,747 (76.6)
	Yes	799 (23.7)	533 (23.4)
**Disposal of baby stools**	Not applicable	609 (18.1)	414 (18.2)
	Safe disposal	1,667 (49.4)	1,138 (49.9)
	Unsafe disposal	1,095 (32.5)	728 (31.9)
**Source of drinking water, dry season**	Safe (village pump, protect well, pipe)	2,322 (68.9)	1,588 (69.7)
	Unsafe (river, pond, canal, rain)	1,049 (31.1)	692 (30.3)
**Walking distance to drinking water source (min)**	≤4	1,640 (48.7)	1,091 (47.9)
	5–9	887 (26.3)	603 (26.4)
	≥10 min	844 (25.0)	586 (25.7)

Almost half of the participants (42.1%) declared consuming raw or undercooked fish (Table 1). This food consumption habit was significantly more frequent among the Lao Loum (45.2%), than among the members of other ethnic groups (28.2%, χ^2^ = 59.53, p<0.001). Fermented fish dishes were widely consumed (74.1%), particularly by the Lao Loum (75.7%) and significantly less by Lao Theung (Lao from the midlands) or the Lao Soung (Lao from the mountains, 67.2%, χ^2^ = 18.84, p<0.001).

### *Opisthorchis viverrini* Infection

**Figure 1:**
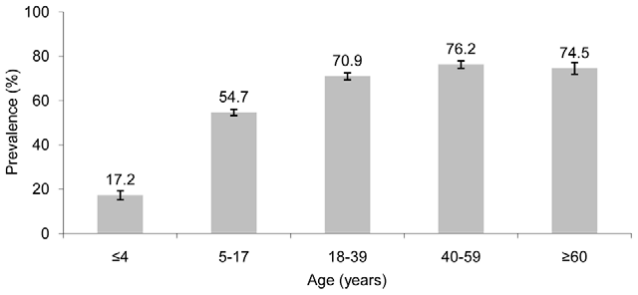
Prevalence of *O. viverrini* infection in Champasack province, southern Lao PDR, stratified by age group. Data were obtained from cross-sectional surveys carried out in 51 villages of Champasack province between January and May 2007.

The overall prevalence of *O. viverrini* infection was 61.1% (95% confidence interval (CI): 59.5–62.8%) with a range from 0% to 95.1% at the unit of the village. Almost three-quarter of the surveyed villages (37/51) had prevalence rates above 50%. The average prevalence of *O. viverrini* infection in villages located at altitudes of 500 m above sea level and higher was 12.8% (95% CI: 9.6–16.0%). In one village in the highlands, no *O. viverrini* infection was found. The average prevalence of *O. viverrini* infection in villages situated at altitudes below 500 m above sea level was 67.9% (95% CI: 66.2–69.6%). Rice farmers were at highest risk of infection (81.3%, 95% CI: 79.2–83.4%), followed by workers of the tertiary sector (73.8%, 95% CI: 64.6–83.1%). With regard to age, the lowest prevalence was observed in children aged ≤4 years with a rapid increase thereafter. Prevalence rates stratified by age group are shown in Figure 1.

### Associations with *O. viverrini* Infection

Results of the bivariate non-spatial logistic regressions, together with category frequencies when applicable, are presented in Table S3. Proportions of environmental covariates that were included in the multivariate model only are displayed. The only covariates found not to be significantly associated with infection, were sex (p = 0.823), the mean annual RFE (p = 0.961), and yearly cumulative RFE (p = 0.954).

### Spatial Analysis of *O. viverrini* Infection Risk

The goodness of model fit, as expressed by the DIC, for all non-spatial and spatial multivariate models are shown in Table S4. Without exception, spatial models resulted in lower DICs, and hence fitted the data better than their non-spatial counterpart models. The model parameters of five spatial multivariate models are summarized in Table 2.

**Table 2:** Model parameters of the five spatially explicit models.

	Model 1		Model 2		Model 3		Model 4		Model 5	
Covariates	No covariates		Environmental		Questionnaire data		Both		Both+treatment	
Model parameters				95% CI		95% CI		95% CI		95% CI
DIC	3,708		3,710		3,157		3,157		2,147	
σ (mean)	3.2	(0.05–7.99)	0.90	(0.46–1.79)	3.62	(1.36–9.94)	1.22	(0.67–2.21)	0.986	(0.52–1.71)
σ (median)	2.72		0.82		2.97		1.14		0.941	
ρ (mean)	3.08	(0.05–6.92)	109.4	(4.23–282)	5.61	(1.33–11.9)	134.6	(7.29–285.8)	156.7	(21.4–287)
ρ (median)	2.77		88.73		4.2		130.6		157.8	
Range mean (deg)	1.22	(0.02–2.79)	0.13	(0.01–0.71)	0.84	(0.25–2.25)	0.06	(0.01–0.41)	0.03	(0.01–0.14)
Range median (deg)	1.08		0.034		0.71		0.02		0.019	
Range median (km)	116.5		3.67		76.3		2.48		2.05	

CI, credible interval; DIC, deviance information criterion (a measure of model fit; a lower DIC indicates a better fit).

Model 1: Bayesian geostatistical model with no covariates; Model 2: Bayesian geostatistical model with environmental covariates only; Model 3: Bayesian geostatistical model with questionnaire derived covariates only; Model 4: Bayesian geostatistical model with environmental and questionnaire-derived covariates, without treatment; Model 5: Bayesian geostatistical model with environmental and questionnaire-derived covariates, including treatment.

σ is the location-specific unexplained variance.

ρ is the decay parameter. The range (range = 3/ρ) is the distance at which the spatial correlation becomes less than 5%.

The data exhibited spatial correlation at a distance up to 116.5 km, as indicated by the range of the model without covariates (model 1). The range dropped to 3.67 km and the sigma parameter was reduced by 63.6% after introduction of environmental covariates. In the presence of questionnaire-derived demographic data, socioeconomic and behavioral factors, the range was 73.6 km and sigma remained comparable to that found without any covariates.

### Subsample for Adjustment for Treatment

In addition to the total sample of 3,371 individuals, Table 1 summarizes the characteristics of those 2,280 individuals who provided information on recent treatment history. The prevalence of *O. viverrini* infection in this subsample was 61.6% (95% CI: 59.5–63.5%), and hence almost identical to the overall prevalence in the full sample (i.e., 61.1%). Given that other demographic, behavioral, and socioeconomic characteristics were similar, model outcomes derived from this subsample were generalized to the total sample.

Results from the non-spatial model (i.e., model 4b) and the two spatially explicit multivariate models that include both questionnaire-derived and environmental data without (model 4) and with (model 5) adjustment for treatment are summarized in Table 3. Taking into account spatial correlation changed the significance of numerous covariates. However, all age categories, and main occupation (i.e., rice farmer, animal breeder and fisherman), consumption of raw or undercooked fish, and mean rainfall over the dry season remained significant after the introduction of the spatial random effect (models 4 and 4b).

**Table 3:** Results for three multivariate models, including questionnaire-derived and environmental covariates.

Covariate		Model 4b		Model 4		Model 5	
		Non-spatial		Spatial		Spatial	
		OR	95% CI	OR	95% CI	OR	95% CI
**Age (years)**	≤4	1.00		1.00		1.00	
	5–17	**5.07**	(3.36–7.52)	**6.57**	(4.29–10.16)	**6.75**	(4.09–11.25)
	18–39	**7.62**	(4.76–11.98)	**11.28**	(6.89–18.95)	**15.24**	(8.36–28.02)
	40–59	**8.90**	(5.47–14.24)	**13.96**	(8.34–24.02)	**18.01**	(9.69–33.85)
	≥60	**15.17**	(9.58–24.24)	**23.01**	(14.00–38.90)	**26.98**	(14.79–50.00)
**Ethnic group**	Other	1.00		1.00		1.00	
	Lao Loum	0.89	(0.66–1.19)	1.56	(0.94–2.58)	**2.25**	(1.28–3.88)
**Socioeconomic status**	Most poor	1.00		1.00		1.00	
	Very poor	**0.68**	(0.51–0.90)	0.90	(0.65–1.25)	0.99	(0.66–1.48)
	Poor	1.23	(0.90–1.68)	**0.60**	(0.43–0.83)	0.77	(0.5–1.13)
	Less poor	0.84	(0.58–1.22)	1.01	(0.71–1.42)	1.09	(0.71–1.65)
	Least poor	**2.21**	(1.68–2.89)	0.81	(0.53–1.22)	1.12	(0.68–1.85)
**Education level**	Illiterate	1.00		1.00		1.00	
	Primary school	1.15	(0.87–1.53)	1.29	(0.95–1.74)	1.37	(0.95–1.96)
	Secondary school and up	1.32	(0.94–1.87)	**1.54**	(1.06–2.23)	**1.64**	(1.05–2.53)
**Main occupation**	No occupation	1.00		1.00		1.00	
	School pupil	**1.50**	(1.04–2.16)	1.38	(0.94–2.03)	1.52	(0.95–2.44)
	Tertiary sector	1.77	(0.95–3.38)	1.80	(0.91–3.61)	1.51	(0.68–3.43)
	Rice farmer	**2.60**	(1.84–3.73)	**2.32**	(1.58–3.41)	**2.40**	(1.56–3.75)
	Raise animals/Fisher/Other	**2.25**	(1.39–3.69)	**2.22**	(1.34–3.76)	**2.21**	(1.20–4.05)
**Consumption of raw or undercooked fish**	No	1.00		1.00		1.00	
	Yes	**1.37**	(1.08–1.75)	**1.36**	(1.10–1.68)	1.23	(0.95–1.59)
**Consumption of fermented fish**	No	1.00		1.00		1.00	
	Yes	**0.76**	(0.63–0.93)	0.96	(0.77–1.21)	1.07	(0.82–1.42)
**Drink bottled or boiled drinking water**	No	1.00		1.00		1.00	
	Yes	0.99	(0.79–1.24)	**0.75**	(0.60–0.95)	0.79	(0.59–1.06)
**Source of drinking water, dry season**	Safe	1.00		1.00		1.00	
	Unsafe	0.87	(0.68–1.10)	1.41	(0.98–2.02)	**1.58**	(1.04–2.40)
**Walk distance to water source, dry season**	≤4 minutes	1.00		1.00		1.00	
	5–9 minutes	**0.72**	(0.54–0.97)	1.18	(0.91–1.52)	1.12	(0.81–1.54)
	≥10 minutes	1.17	(0.92–1.48)	1.12	(0.83–1.52)	1.14	(0.79–1.65)
**Access to toilets**	No	1.00		1.00		1.00	
	Yes	1.07	(0.82–1.38)	0.74	(0.54–1.01)	**0.57**	(0.38–0.84)
**Disposal of baby stools**	Not applicable	1.00		1.00		1.00	
	Safe disposal	**1.38**	(1.15–1.66)	1.25	(0.96–1.61)	1.32	(0.95–1.82)
	Unsafe disposal	0.90	(0.73–1.10)	1.00	(0.74–1.35)	1.02	(0.7–1.48)
**Treatment**	No treatment					1.00	
	Traditional medicine					1.23	(0.86–1.80)
	Praziquantel					**0.35**	(0.20–0.63)
	Other chemotherapy					1.03	(0.73–1.44)
**LST day**		**1.43**	(1.22–1.67)	1.48	(0.88–2.56)	1.43	(0.84–2.38)
**LST night**		**0.44**	(0.29–0.65)	0.62	(0.16–2.31)	0.65	(0.19–2.27)
**Rainfall (RFE means)**		**0.75**	(0.67–0.84)	**0.67**	(0.45–0.97)	0.78	(0.54–1.11)
**EVI**		**1.17**	(1.02–1.34)	1.13	(0.7–1.82)	1.26	(0.8–2.01)
**Altitude**		**0.996**	(0.995–0.997)	1.00	(0.99–1)	1.00	(0.99–1.00)
**Land use/land cover**	Savanna, grass, shrubs	1.00		1.00		1.00	
	Water and wetlands	1.16	(0.86–1.56)	1.19	(0.43–3.36)	1.00	(0.35–2.75)
	Forest	0.76	(0.51–1.13)	0.67	(0.17–2.58)	0.80	(0.21–2.95)
	Cropland, bare & built soil	**0.66**	(0.52–0.84)	0.76	(0.33–1.74)	0.79	(0.36–1.78)
**Distance to water bodies (km)**		**0.85**	(0.81–0.89)	0.84	(0.70–1.01)	**0.80**	(0.67–0.97)

CI, credible interval; EVI, enhanced vegetation index; LST, land surface temperature; OR, odds ratios (posterior median); RFE, rainfall estimate.

OR in bold are significant at 5% level, as indicated by the Bayesian credible interval; Model 4: Bayesian geostatistical model with environmental and questionnaire-derived covariates; Model 4b: same covariates as Model 4 but without the geostatistical component (non spatial model); Model 5: similar to Model 4, plus treatment.

### Risk Factors for *O. viverrini* Infection

The spatial model with adjustment for treatment (i.e., model 5) was the best fitting one, as it had the lowest DIC. Hence, this model was utilized for subsequent identification of risk factors for *O. viverrini* infection. Similar to the spatial model without treatment adjustment (model 4), increasing age (5–17 years: odds ratio (OR) = 6.75 (95% CI: 4.09–11.25); 18–39 years: OR = 15.24 (95% CI: 8.36–28.02); 40–59 years: OR = 18.01 (95% CI: 9.69–33.85); and ≥60 years: OR = 26.98 (95% CI: 14.79–50)), at least secondary school attainment (OR = 1.64, 95% CI: 1.05–2.53), main occupation (rice farmer (OR = 2.40, 95% CI: 1.56–3.75); raising animals or fisherman (OR = 2.21, 95% CI: 1.20–4.05)) were risk factors for infection.

Adjusting for praziquantel treatment history changed the significance of several explanatory factors. For example, boiled or bottled drinking water, rainfall and, most importantly, consumption of raw or undercooked freshwater fish showed no significance any longer. Access to sanitation turned out to be a protective factor (OR = 0.57, 95% CI: 0.38–0.84), whereas drinking water from unsafe sources (OR = 1.58, 95% CI: 1.04–2.40) and Lao Loum ethnicity (OR = 2.25, 95% CI: 1.28–3.88) were risk factors. The risk decreased by two-third (OR = 0.35, 95% CI: 0.20–0.63) in case of prior treatment with praziquantel. The only significant environmental parameter was distance to freshwater bodies, with a 20% risk reduction for each additional km away from large water bodies (OR = 0.80, 95% CI: 0.67–0.97).

### Spatial Prediction of Infection Risk

**Figure 2:**
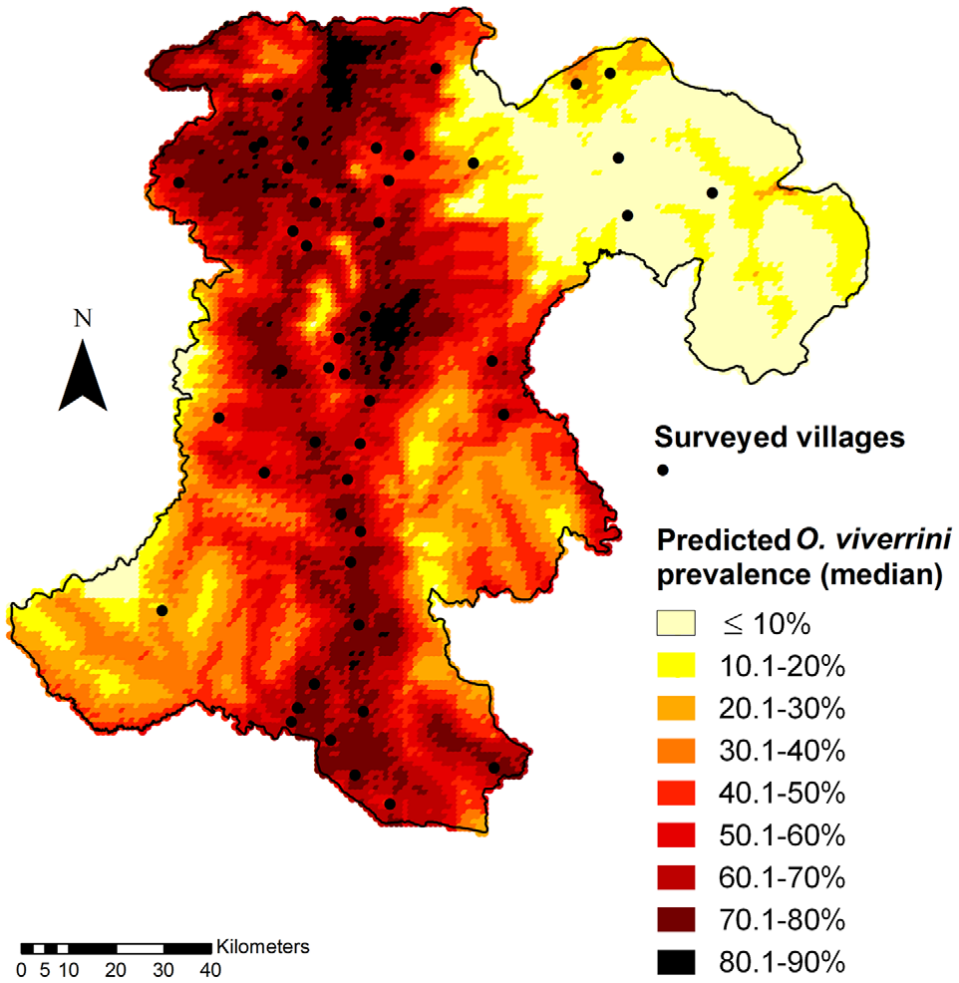
Map of the predicted prevalence (median) of *O. viverrini* infection in Champasack province, southern Lao PDR.

Table 4 presents the OR for covariates used in the predictive model. Figure 2 displays the predicted median prevalence of *O. viverrini* in Champasack province, southern Lao PDR. Low prevalence rates were predicted at altitudes of at least 500 m and up to 1,617 m above sea level. Our results are consistent with the observed prevalence in villages of these areas.

**Table 4:** Odds ratio and significance of environmental covariates in the predictive model (Model 2).

Covariate		Model 2 (predictive model)	
		OR	95% CI
**LST day**		1.32	(0.85–2.07)
**LST night**		1.09	(0.36–3.82)
**Rainfall (RFE means)**		**0.69**	(0.48–0.96)
**EVI**		1.10	(0.74–1.65)
**Altitude**		1.00	(0.99–1.00)
**Land use/land cover**	Savanna, grass, shrubs	1.00	
	Water and wetlands	1.01	(0.44–2.44)
	Forest	0.77	(0.24–2.38)
	Cropland, bare & built soil	0.79	(0.40–1.54)
**Distance to water bodies (km)**		**0.84**	(0.72–0.99)

CI, credible interval; EVI, enhanced vegetation index; LST, land surface temperature; RFE, rainfall estimate; OR, odds ratios.

OR in bold are significant at 5% level, as indicated by the Bayesian credible interval.

**Figure 3:**
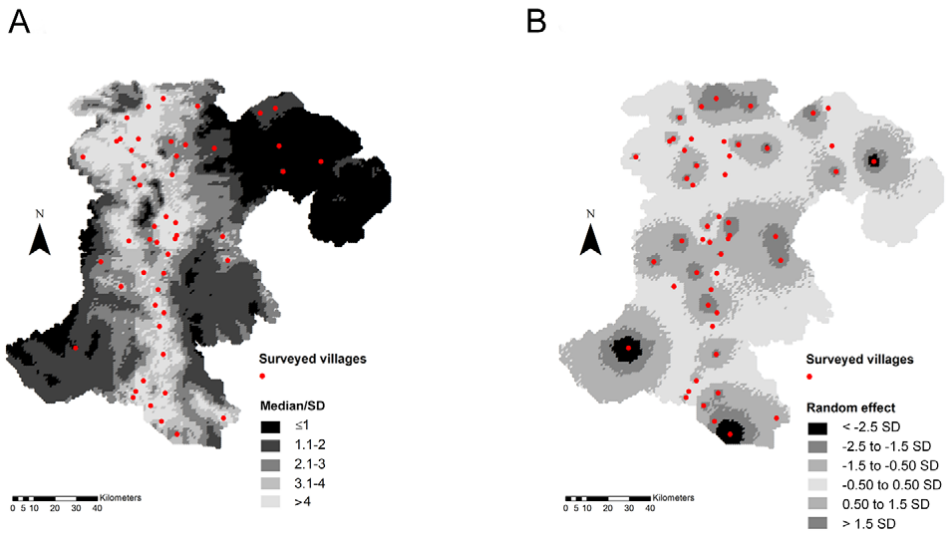
Error coefficient of the predicted *O. viverrini* prevalence (a) and location-specific random effects (b). The error coefficient is the ratio of the predicted prevalence median over its standard deviation (SD). Darker zones indicate higher uncertainty of the predictive model.

Plotted in Figure 3, the error coefficient (3a) is the ratio of the predicted prevalence median over its standard deviation, and is a measure of model uncertainty. The random effects (3b) represent the deviation between the mean observed and predicted prevalence rates. A lower value of this ratio corresponds to a lower precision of the prediction. The distributions of the environmental covariates, together with the river network in the Champasack province, are presented in Figure S1.

### Model Validation

**Figure 4:**
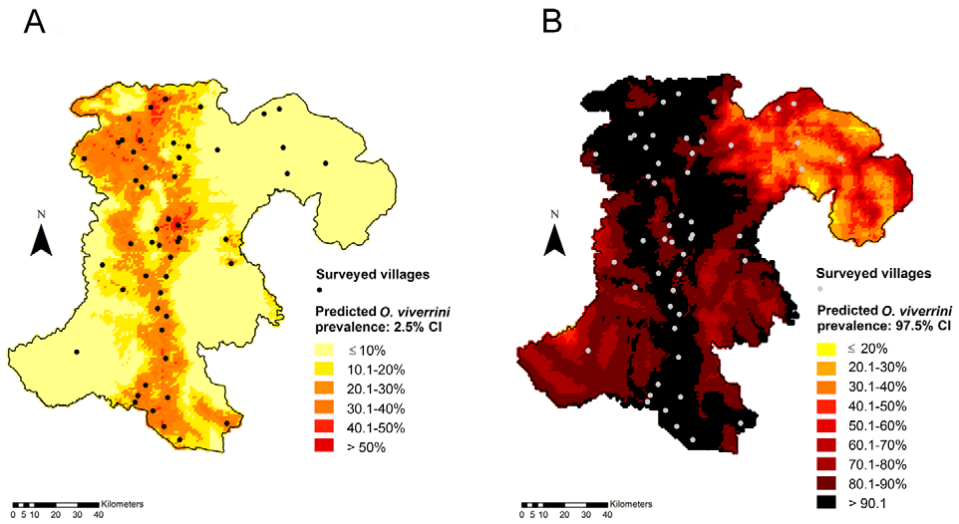
Lower (a) and upper estimates (b) of the predicted prevalence of *O. viverrini* infection in Champasack province, southern Lao PDR. The lower and upper estimates pertain to the 2.5% and 97.5% confidence interval (CI), respectively.

Our final model was able to predict 100% of the test locations and the *O. viverrini* prevalence estimates within an 80% credible interval. After removing 10% of the locations to fit the model for validation, the significance of rainfall disappeared. Figure 4 shows the lower (4a) and upper (4b) credible interval limits for the predicted prevalence. Those values correspond to the minimum and maximum estimates for *O. viverrini* prevalence at each of the non-sampled location.

## Discussion

Microscopic examination of duplicate Kato-Katz thick smears derived from a single stool sample revealed a prevalence of *O. viverrini* of over 60% in a random sample of more than 3,000 individuals from 51 villages confirming the high endemicity of this liver fluke infection in Champasack province, southern Lao PDR. Two limitations of the Kato-Katz technique need consideration. First, the differential diagnosis of *O. viverrini* and MIF eggs is exceedingly difficult. It is therefore conceivable that some of the *O. viverrini* infections reported here were in reality MIF, and hence the reported prevalence of *O. viverrini* might be an overestimate of the true infection prevalence. However, although concurrent infections with *O. viverrini* and MIF are common in Lao PDR, and a recent in-depth study examining adult flukes through purgation found that *O. viverrini*-infected individuals were indeed frequently co-infected with MIFs, single MIF infection were very rare [5], [24]. It follows that individuals considered *O. viverrini*-positive were likely to be infected with this parasite species, and perhaps some co-infected with MIF. Second, due to the imperfect sensitivity of the Kato-Katz technique, the true *O. viverrini* prevalence might be considerably higher than reported here [36]. Preceding studies have indeed shown that additional Kato-Katz thick smears from multiple stool samples or the concurrent use of different diagnostic methods (e.g., Kato-Katz plus formalin-ethyl acetate concentration technique) result in higher prevalence estimates of *O. viverrini* and other helminth infections [9], [37], [38]. Despite these limitations, our findings are in line with previous observations of high infection prevalence of *O. viverrini* in Saravane and Khammouane provinces.

Helminth prevalence estimates are affected by control measures. In this connection it is interesting to note that large-scale administration of anthelmintic drugs was interrupted in Champasack province in the late 1990s and only resumed in 2008, after our study had been implemented [27]. This might explain the low treatment coverage, as in our study cohort, only 3.5% reported having received praziquantel.

Another shortcoming of our study is that no attempt was made to quantify infection intensity. Given the large number of stool samples to be examined in more than 50 sites with a small team and limited financial resources, it was operationally not feasible to count all the helminth eggs in the Kato-Katz thick smears. Moreover, there is the challenge of differential diagnosis between *O. viverrini* and MIF as explained before.

After adjusting for praziquantel treatment history, which in addition to the introduction of spatial correlation had important implications both in terms of model fit and significance of covariates, the following key determinants of *O. viverrini* infection were identified. The risk of infection increased with age, higher educational attainment (i.e., secondary schooling and above), rural occupation (rice farmer, fisherman, and animal breeder), use of unsafe drinking water, and ethnicity (Lao Loum were at higher risk of infection). On the other hand, access to sanitation, living further away from large freshwater bodies, and history of praziquantel treatment were found to be protective factors.

With regard to age, the risk of *O. viverrini* infection gradually increased during childhood, reaching a plateau among adolescence, young adults, and the elderly [8], [39]. A similar age-prevalence pattern has been observed for *O. viverrini* in Thailand [39], [40]. Although the prevalence of *O. viverrini* in children was considerably lower than their older counterparts, people of all ages are at risk. Worryingly, the youngest individual found with *O. viverrini* eggs in the stool was as young as 6 months. Early weaning and substitution with complementary solid food, including fish, is a common habit in rural Lao PDR, and starts within the first months of life [41]. Regarding CCA, which takes 30–40 years to develop, people infected with *O. viverrini* in their childhood may only declare this cancer during their most productive years of life, with far-reaching negative consequences for families and entire communities [42]. There is a need to estimate the global burden of opisthorchiasis and other food-borne trematodiases, including the associated risk of *O. viverrini* infection and CCA [1], [2]. Once these estimates and new evidence becomes available, it should help to further raise awareness of opisthorchiasis and to put forth concerted actions for control of *O. viverrini*, which, despite its undeniable public health importance, remains largely neglected [14], [43].

Surprisingly, our models revealed only very weak associations between infection and consumption of raw or insufficiently cooked fish. Indeed, after adjustment for praziquantel treatment history, this association showed no statistical significance. Over half of the infected individuals (52.1%) declared that they did not consume undercooked fish. However, there might have been some confusion due to infections with lecithodendriid trematodes, which are transmitted by insects rather than fish. It is difficult to distinguish eggs of lecithodendriid and *O. viverrini* in Kato-Katz thick smears. Nevertheless, we believe that lecithodendriid single infections are rare, similar to previous observations with MIF single infections in the current study area [24]. Contamination of food, hands, and surfaces on food preparation utensils with the infective stage of the parasite (i.e., metacercariae) may be responsible for some of the infections [39]. Although the bulk of metacercariae are killed through fermentation and salting, cooking is the most efficient strategy to prevent infection. Indeed, previous research has shown that fermented fish dishes are not entirely safe with regard to transmission of opisthorchiasis [40], [44], although no clear association was found between consumption of such food and infection in multivariate spatial models. It is conceivable that infections arising from rare surviving parasites in fermented food are of light intensity, and hence are likely to be missed by duplicate Kato-Katz thick smears derived from a single stool sample. Still, the respective infectivity of fermented dishes, reported here to be consumed by almost three-quarter of the study participants, should be further assessed. We speculate that consumption of raw fish was under-reported. Probable causes may be a lack of clarity in the term “insufficiently cooked” or stigmatization. Listing fish dishes, investigating their infectivity, and use of their local names in questionnaires could help remedy these issues.

No association was found between infection risk and socioeconomic status despite the use of a robust data reduction technique for categorical asset data [30]. This observation suggests that people from different socioeconomic strata share the same risk of infection, and hence the consumption of those dishes is intimately linked to culture and tradition rather than wealth. As for the surprising association that people with higher education levels are at higher risk of *O. viverrini* infection, this might be explained by the fact that two-third of participants with higher education were rice farmers, fishermen, animal breeders, or tertiary sector workers; occupational groups with the highest prevalence rates.

Similar to observations made in Saravane province, access to traditional latrines and pour flush toilets emerged as a protective factor against *O. viverrini* infection [8]. Given that none of the studied villages had full latrine coverage, sanitation could not impact infection directly but rather had an effect on transmission and infection intensity, through reduction of environmental contamination. Infection with *O. viverrini* does not occur directly through drinking contaminated water, so the positive effect of safe drinking water (and improved sanitation), rather relates to a health-promoting behavior that indirectly improved health status.

Another important finding of our study is the strong spatial heterogeneity of *O. viverrini* at the unit of the village, including village pairs with similar age profiles that are in close proximity to each other. This observation confirms the high focality of *O. viverrini* infection at a small spatial scale [7], [9], [39]. Bayesian methods that explicitly incorporate the spatial structure of the data offer several unique features over classical frequentist approaches. First and foremost, the spatial models employed here more accurately explained the variability of risk compared to their non-spatial counterparts. Second, the significance of most environmental factors disappeared after accounting for spatial correlation. This finding is consistent with the fact that omitting spatial correlation when present might result in inaccurate estimates of covariates regression coefficients, resulting in erroneous conclusion of significance [45].

The dramatic drop of the range and the strong reduction of village-specific variance (sigma) after introduction of environmental covariates both indicate that environmental factors account for most of the spatial correlation. In presence of questionnaire-derived covariates, the range remained high and sigma unchanged, which suggests that there is only minor spatial correlation of demographic, socioeconomic, sanitation, and behavioral factors. Climatic factors are known to delimit suitable living areas for intermediate hosts, thereby conditioning the spatial distribution of parasitic diseases [13], [14]. Lower risk in altitude might relate to local features that do not meet ecological requirements of the first intermediate host snails of the genus *Bithynia* [46]. However, infection risk was significantly associated with distance to water bodies and rainfall amount only, and hence our data do not support an association between ecological requirements of the snails and environmental features. This observation suggests that the role of the environment would rather relate to exposure to water and access to the second intermediate host fish, whereas climatic factors would be too homogeneous at this small spatial scale to influence the distribution of intermediate hosts.

The random selection of survey locations in the study area resulted in an uneven distribution of villages over the different ecological settings of the study province. The lack of sampled locations in the south-west, the centre-east and the mountains affected the predictive ability of the model, and hence uncertainty was higher in those three areas. Sampling should be stratified over ecological zones. Most likely, this issue affected the significance of the relationship with elevation, as the absence of association with other environmental factors is consistent with the homogeneity of climatic conditions at a provincial scale. Our results are therefore consistent with a previous study conducted by Clements and colleagues, who did not find any association between schistosomiasis and environmental factors despite using data specifically collected for spatial analysis [47].

The association between environmental factors and risk was strong enough to clearly delineate between low-risk and a high-risk zones. Indeed, low risks were found at study locations with an altitude of at least 500 m above sea level. This risk difference may not only arise from lower accessibility of suitable freshwater fish, as conditioned by larger distance to water and higher rainfall, but is likely to be also due to human features correlated with altitude. Indeed all but three rice farmers, who were in the occupational group with the highest prevalence, lived in the plains. Moreover, the ethnic composition of mountain villages was dominated by Lao Theung and Lao Soung, the ethnic groups who do not have a strong preference for consumption of raw fish.

Another important feature of Bayesian geostatistical modeling is that it allows assessment of model uncertainty. This not only provides useful indications for methodology, but also prevents relying on imprecise estimations, a crucial aspect when it comes to using risk predictions of neglected tropical diseases to guide subsequent control interventions. The point estimates of predicted prevalence in this study had only a low precision, and hence setting-specific estimates must be interpreted with caution. However, the model was able to correctly predict prevalence at all test locations and, despite large credible intervals of the predictions, the same two different risk zones remained clearly identified by the higher and lower prevalence estimates.

In conclusion, this study provides new insight into the distribution of *O. viverrini* infection in Champasack province and emphasizes the use of environmental factors to predict *O. viverrini* prevalence. It appears that an infection with *O. viverrini* is governed by the interplay of environmental factors underlying the access to second intermediate host fish species, and human behavior. In Champasack province, raw fish consumption is common, particularly among the Lao Loum ethnic group who represents 80% of the population. The role of the environment seems to mainly pertain to freshwater fish accessibility, which, combined with the strong preference of the Lao Loum for raw fish dishes, results in higher risk, whereas the lack of sanitation sustains high transmission in the region. The high prevalence of *O. viverrini* infection in Champasack province is alarming, and calls for urgent public health interventions. Indeed, administration of praziquantel to entire populations should be considered as the first priority, particularly in the Mekong River corridor, to control morbidity. This intervention should be coupled with setting-specific information, education, and communication strategies (avoiding consumption of raw food dishes and improved hygiene), and access to clean water and sanitation.

## Supporting Information

Figure S1 **Distribution of environmental factors in Champasack province, southern Lao PDR.**
(TIF)

Table S1 Temporal and spatial resolutions of the environmental covariates used in the analysis.
(DOC)

Table S2 Nomenclature for spatial and non-spatial models, with and without adjustment for treatment.
(DOC)

Table S3 Results for non spatial bivariate regressions.
(DOCX)

Table S4 Goodness of fit (DIC) of non-spatial and corresponding spatial models.
(DOC)

Appendix S1 Model formulation.
(DOCX)
