# Supplementary figures and images for: Spatial Distribution of, and Risk Factors for, Opisthorchis viverrini Infection in Southern Lao PDR
Source: PLoS Negl Trop Dis. 2012 Feb 14;6(2):e1481. doi: 10.1371/journal.pntd.0001481 (PMC3279336; doi:10.1371/journal.pntd.0001481)

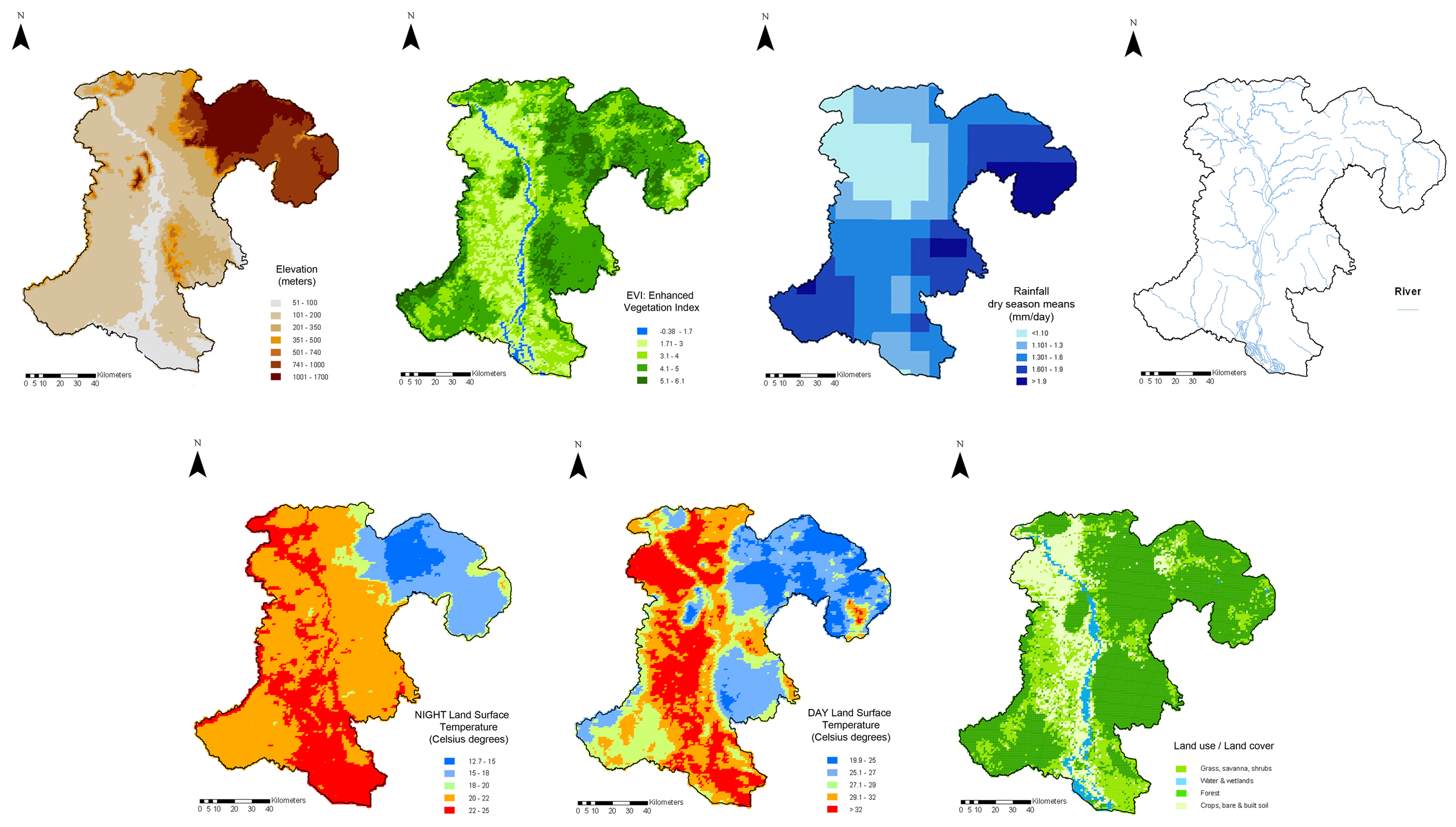

Supplement: Figure S1 — Distribution of environmental factors in Champasack province, southern Lao PDR. (TIF) [file pntd.0001481.s001.tif]
