# Supplementary material for: Spatial Distribution of, and Risk Factors for, Opisthorchis viverrini Infection in Southern Lao PDR
Source: PLoS Negl Trop Dis. 2012 Feb 14;6(2):e1481. doi: 10.1371/journal.pntd.0001481 (PMC3279336; doi:10.1371/journal.pntd.0001481)
Supplement: Table S1 — Temporal and spatial resolutions of the environmental covariates used in the analysis. (DOC) [file pntd.0001481.s002.doc]

| **Covariate** | **Temporal resolution** | **Spatial resolution** | **Time frame** | **Source** |
| --- | --- | --- | --- | --- |
| EVI | Monthly | 1x1 km | Jan 2006 - May 2007 | MODIS |
| Day & Night LST | 8 days | 1x1 km | Jan 2006 - May 2007 | MODIS |
| LULC | Yearly | 1x1 km | 2004 | MODIS |
| RFE | daily | 10x11 km | Jan 2006 - May 2007 | CPC FEWS |
| Altitude | - | 90x90 m | - | SRTM CGIAR-CSI |
| Distance to water | - | - | - | Health Mapper |
