# Supplementary material for: Spatial Distribution of, and Risk Factors for, Opisthorchis viverrini Infection in Southern Lao PDR
Source: PLoS Negl Trop Dis. 2012 Feb 14;6(2):e1481. doi: 10.1371/journal.pntd.0001481 (PMC3279336; doi:10.1371/journal.pntd.0001481)
Supplement: Table S2 — Nomenclature for spatial and non-spatial models, with and without adjustment for treatment. (DOC) [file pntd.0001481.s003.doc]

| **Covariates** | **No covariates** | **Environmental** | **Questionnaire** | **Both** | **Both + Treatment** |
| --- | --- | --- | --- | --- | --- |
| Sample size | n = 3,371 | n = 3,371 | n = 3,371 | n = 3,371 | n = 2,280 |
| Spatial model | Model 1 | Model 2 | Model 3 | Model 4 | Model 5 |
| Non spatial model | - | Model 2b | Model 3b | Model 4b | Model 5b |
