# Supplementary material for: Spatial Distribution of, and Risk Factors for, Opisthorchis viverrini Infection in Southern Lao PDR
Source: PLoS Negl Trop Dis. 2012 Feb 14;6(2):e1481. doi: 10.1371/journal.pntd.0001481 (PMC3279336; doi:10.1371/journal.pntd.0001481)
Supplement: Table S3 — Results for non spatial bivariate regressions. (DOCX) [file pntd.0001481.s004.docx]

| **Variable** | **Category** | **% of sample** | **OR** | **95% CI** | **LRT**  **p-value** |
| --- | --- | --- | --- | --- | --- |
| **Sex** | 0. Female | 52.68 | 1.00 |  | 0.8225 |
|  | 1. Male | 47.32 | 0.07 | 0.86 , 1.13 |  |
| **Ethnic group** | 0. Other | 18.27 | 1.00 |  | <0.001 |
|  | 1. Lao Loum | 81.73 | 4.21 | 3.50 , 5.07 |  |
| **Age (years)** | 0. 0-4 | 9.64 | 1.00 |  | <0.001 |
|  | 1. 5-17 | 36.64 | 5.81 | 4.27 , 7.91 |  |
|  | 2. 18-39 | 26.31 | 11.71 | 8.48 , 16.16 |  |
|  | 3. 40-59 | 19.25 | 15.44 | 10.99 , 21.69 |  |
|  | 4. ≥ 60 | 8.16 | 14.07 | 9.47 , 20.89 |  |
| **Main occupation** | 0. No occupation | 21.71 | 1.00 |  | <0.001 |
|  | 1. School pupil | 26.61 | 2.08 | 1.70 , 2.53 |  |
|  | 2. University/Employee/Business | 2.61 | 4.62 | 2.80 , 7.60 |  |
|  | 3. Rice farmer | 39.72 | 7.11 | 5.81 , 8.71 |  |
|  | 4. Fisherman/Raise animals/Other | 9.34 | 1.10 | 0.84 , 1.45 |  |
| **Socioeconomic status** | 1. Most poor | 17.26 | 1.00 |  | <0.001 |
|  | 2. Very poor | 19.49 | 0.86 | 0.68 , 1.07 |  |
|  | 3. Poor | 21.92 | 0.81 | 0.65 , 1.01 |  |
|  | 4. Less poor | 21.21 | 1.39 | 1.11 , 1.75 |  |
|  | 5. Least poor | 20.11 | 1.13 | 0.90 , 1.42 |  |
| **Education level** | 0. Illiterate | 28.63 | 1.00 |  | <0.001 |
|  | 1. Primary school | 52.42 | 2.19 | 1.87 , 2.57 |  |
|  | 2. Secondary school and up | 18.96 | 2.67 | 2.16 , 3.3 |  |
| **Consumption of raw or undercooked fish** | 0. No | 57.88 | 1 |  | <0.001 |
|  | 1. Yes | 42.12 | 1.87 | 1.62 , 2.16 |  |
| **Consumption of fermented fish** | 0. No | 25.87 | 1.00 |  | <0.001 |
|  | 1. Yes | 74.13 | 1.56 | 1.33 , 1.82 |  |
| **Bottled or boiled drinking water** | 0. No | 45.03 | 1.00 |  | 0.0046 |
|  | 1. Yes | 54.97 | 0.82 | 0.71 , 0.94 |  |
| **Access to toilets** | 0. No | 76.30 | 1 |  |  |
|  | 1. Yes | 23.70 | 1.32 | 1.12 , 1.56 | |
| **Disposal of baby stools** | 0. Not applicable | 18.07 | 1.00 |  | <0.001 |
|  | 1. Safe disposal | 49.45 | 0.64 | 0.52 , 0.78 |  |
|  | 2. Unsafe disposal | 32.48 | 0.43 | 0.35 , 0.54 |  |
| **Source of drinking water, dry season** | 0. Safe | 68.88 | 1.00 |  | <0.001 |
|  | 1. Unsafe | 31.12 | 1.58 | 1.36 , 1.84 |  |
| **Walking distance to drinking water source** | 0.≤ 4 | 48.65 | 1.00 |  | 0.0249 |
| **(min)** | 1. 5 to 9 | 26.31 | 1.26 | 1.06 , 1.49 |  |
|  | 2. ≥ 10 | 25.04 | 1.08 | 0.91 , 1.28 |  |
| **Environment** |  |  |  |  |  |
| **LST day, dry season mean** |  |  | 1.24 | 1.20 , 1.27 | <0.001 |
| **LST night, dry season mean** |  |  | 1.41 | 1.35 , 1.46 | <0.001 |
| **EVI, dry season mean** |  |  | 0.61 | 0.57 , 0.65 | <0.001 |
| **Rainfall, dry season mean** |  |  | 0.26 | 0.21 , 0.32 | <0.001 |
| **Altitude** |  |  | 0.997 | 0.996 , 0.997 | <0.001 |
| **Land Use / Land cover** | 0. Savannah, grass, shrubs | 34.32 |  |  | <0.001 |
|  | 1. Water and wetlands | 20.56 | 1.73 | 1.41 , 2.12 |  |
|  | 2. Forest | 12.13 | 0.22 | 0.17 , 0.29 |  |
|  | 3. Cropland, bare and built soil | 32.99 | 1.29 | 1.08 , 1.53 |  |
| **Distance to large water bodies (km)** |  |  | 0.82 | 0.79 , 0.84 | <0.001 |
