# Supplementary material for: Spatial Distribution of, and Risk Factors for, Opisthorchis viverrini Infection in Southern Lao PDR
Source: PLoS Negl Trop Dis. 2012 Feb 14;6(2):e1481. doi: 10.1371/journal.pntd.0001481 (PMC3279336; doi:10.1371/journal.pntd.0001481)
Supplement: Table S4 — Goodness of fit (DIC) of non-spatial and corresponding spatial models. (DOC) [file pntd.0001481.s005.doc]

| **Covariates** | **Environmental only** | **Questionnaire only** | **Both** | **Both + Treatment** |
| --- | --- | --- | --- | --- |
| Spatial | Model 2 | Model 3 | Model 4 | Model 5 |
| DIC | 3709.63 | 3156.57 | 3157.46 | 2147.22 |
| Non spatial | Model 2b | Model 3b | Model 4b | Model 5b |
| DIC | 3974.43 | 3680.31 | 3407.53 | 2296.43 |
