# Supplementary material for: Spatial Distribution of, and Risk Factors for, Opisthorchis viverrini Infection in Southern Lao PDR
Source: PLoS Negl Trop Dis. 2012 Feb 14;6(2):e1481. doi: 10.1371/journal.pntd.0001481 (PMC3279336; doi:10.1371/journal.pntd.0001481)
Supplement: Appendix S1 — Model formulation. (DOCX) [file pntd.0001481.s006.docx]

**Appendix S1:** Bayesian Model Formulation

We assumed the infection status Y*_ij_* of an individual *i* at a village *j* followed a Bernouilli distribution: Y_ij_ ~ Be (p*_ij_*) where p*_ij_* is the probability of being infected by *O. viverrini* for an individual *i* in a village *j*. Spatial random effects *φ*_j_, accounted for unobserved spatial processes at every village *j*. Covariates β*_k_* and random effects *φ*_j_ were modelled on a log *it* scale: $\log it\left(p_{ij}\right)=\alpha+\sum_{k=1}^{n} \beta_{k}X_{kij}+\varphi_{j}$, with *n* being the number of covariates. The random effect $\varphi_{-} =(\varphi_{1},\varphi_{2},.....,\varphi_{j})^{T}$ is assumed to follow a Normal distribution $\varphi_{-} \sim N(0,\Sigma)$, where Σ is the covariance matrix. A stationary isotropic process was assumed in the present work, with the covariance matrix $\Sigma_{ij}=\sigma^{2}corr_{ij}(d_{ij},\rho)$ and an exponential correlation function $corr_{ij}(d_{ij},\rho)=\exp(-d_{ij},\rho)$where *d_ij_* is the shortest distance between two locations *s_i_* and *s_j_*, and ρ is a measure of how spatial correlation decreases with the distance. The distance at which the spatial correlation between villages gets under 5% is equal to 3/ρ and is called the range.

According to Bayesian modelling specification, we chose prior distributions for all parameters to be estimated. We chose a Normal distribution with a mean of zero and a variance of 100 for the regression coefficients. A non informative inverse gamma vague prior with mean equal to 1 and variance equal to 100 was adopted for σ^2^, and a uniform prior for ρ with parameters calculated as a function of the minimum and maximum distance between sampled villages:

$\rho\sim dunif(-\frac{\log(0.01)}{\min\text{distance}};-\frac{\log(0.01)}{\max\text{distance}})$.

A double-chain sampler was run for all models, with a burn-in of 3,000 for non spatial models, and a burn-in of 5,000 for spatial models. Convergence was assessed through examination of the ergodic averages of selected parameters. Before drawing samples, it was verified for each parameter that the MC error was below 5% of the standard deviation. Convergence was reached after 20,000 iterations for non spatial models and after 50,000 iterations for spatial models.

Autocorrelation was checked by visual assessment of the WinBUGS autocorrelation function. Important autocorrelation was found for the intercept and some of the regression coefficients. A thinning of 10 was used to reduce autocorrelation before drawing samples.
